# Combined Cognitive and Psychological Interventions Improve Meaningful Outcomes after Acquired Brain Injury: A Systematic Review and Meta-Analysis

**DOI:** 10.1007/s11065-023-09625-z

**Published:** 2023-11-13

**Authors:** Alexandra Davies, Jeffrey M. Rogers, Katharine Baker, Lily Li, Joshua Llerena, Roshan das Nair, Dana Wong

**Affiliations:** 1 School of Psychology & Public Health, La Trobe University, Bundoora, VIC 3086 Australia; 2 Faculty of Health Sciences, University of Sydney, Camperdown, NSW 2006 Australia; 3 School of Medicine, University of Nottingham, University Park, Nottingham, NG7 2RD UK; 4 Health Research, SINTEF Digital, Dept. of Health Research, Torgaarden, P.O. Box 4760, Trondheim, NO-7465 Norway

**Keywords:** Acquired brain injury, Combined intervention, Cognition, Emotion, Rehabilitation, Cognitive rehabilitation, Psychological therapy

## Abstract

Interventions addressing cognitive and emotional difficulties after acquired brain injury (ABI) often focus on specific impairments in cognition or mood. These interventions can be effective at addressing their specific target, but do not routinely translate to improved activity and participation outcomes. Approaches that combine cognitive and psychological rehabilitation are increasingly popular; however, to date, there have been no systematic evaluations of their efficacy. We conducted a systematic review of five databases, searching for randomised controlled trials of adults with diagnoses of non-progressive ABI at least 1-month post injury, in receipt of interventions that combined cognitive and psychological components compared to any control. Screening and data extraction were evaluated by two independent reviewers using a standardised protocol. Effect sizes were calculated using Hedge’s *g* and estimated using a random-effects model. Risk of bias was assessed using the PEDro-P rating system, and quality of evidence evaluated using the grading of recommendation, assessment, development and evaluation (GRADE) approach. Thirteen studies were included in the meta-analysis (*n* = 684). There was an overall *small*-to-*medium* effect (*g* = 0.42) for combined interventions compared with controls, with gains maintained at 6-month follow-up. Improvements were observed at the level of impairment, activity, participation and quality of life. GRADE ratings and analyses investigating sensitivity, heterogeneity and publication bias indicated that these effects were robust. No a priori variables moderated these effects. Overall, this review provides strong evidence that combined cognitive and psychological interventions create meaningful change in the lives of people with ABI.

Acquired brain injury (ABI) is a leading cause of disability worldwide, placing a significant burden on survivors, caregivers, and healthcare systems (e.g. Access Economics, 2009; Deloitte Access Economics, 2013). The majority of survivors face pervasive and enduring changes to their psychological and cognitive functioning, including clinically significant levels of depressive and anxiety symptoms (Anson & Ponsford, 2006; Campbell Burton et al., 2013; Hackett & Pickles, 2014) and impairments in attention, memory and executive function (Mellon et al., 2015; Rabinowitz & Levin, 2014). Irrespective of injury severity, these changes are associated with poorer functional outcomes (Stolwyk et al., 2021) and are identified as long-term unmet needs by survivors (Andrew et al., 2014). Thus, understanding which interventions best address these unmet needs is critical in order to minimise both the economic and human cost of ABI.

To optimise rehabilitation outcomes, it is important to consider the consequences of ABI in a holistic manner that is meaningful to the individual. The International Classification of Functioning (ICF) provides a conceptual framework for understanding disability (World Health Organisation [WHO], 2002). In doing so, it not only provides a common language for researchers and clinicians to understand and evaluate interventions, but also reflects changes that are important to the ABI survivor. The model describes the effect of disability on an individual at different levels: ‘impairment’ refers to loss or reduced ability in body function or structure (e.g. impairment on memory tests), ‘activity limitation’ refers to the difficulties a person may have in executing activities (e.g. forgetting appointments), and ‘participation restriction’ refers to the difficulties a person may experience with participating in meaningful life roles (e.g. not being able to work as a result of memory difficulties) (WHO, 2002). Another important level to consider is the concept of quality of life, which is not included in the ICF framework. Though intervention outcomes are often measured at the impairment level, outcomes at the activity, participation and quality of life levels best reflect meaningful change in a person’s life and are the most common focus of survivors.

Cognitive and psychological interventions evaluated in clinical trials post-ABI are usually highly targeted, focusing on a specific cognitive domain or mood disturbance. Psychological interventions use therapies such as cognitive behaviour therapy (CBT) or associated strategies (O'Malley et al., 2016) to address difficulties with emotions, thoughts or behaviour (Hickey et al., 2019). Cognitive rehabilitation refers to a structured set of therapeutic activities designed to either retrain individual cognitive domains (such as attention and memory) or cognitive functions more generally, or to teach people to compensate for their cognitive deficits with the specific aim of addressing the cognitive problem (Lincoln et al., 2015).

Though these interventions can be effective at addressing their specific target at the impairment level (i.e. mood or cognition), they do not always translate to improved activity, participation and quality of life outcomes. The evidence regarding cognitive interventions is strong at the level of impairment (effect sizes ranging from 0.25 to 0.75, depending on domain) (Cicerone et al., 2019; das Nair et al., 2016; Loetscher et al., 2019; Rogers et al., 2018; Taylor et al., 2021); however, the evidence for benefits at the activity and participation level is less consistent. For example, Loetscher et al. (2019) found that the benefits observed from cognitive rehabilitation targeting attention deficits did not generalise daily life activities or quality of life outcomes, while Taylor et al. (2021) found some quality-of-life differences favouring their memory rehabilitation group compared to controls in a multiple sclerosis population.

For psychological interventions, the evidence is less clear. Two previous meta-analyses found *moderate* to *large* effect sizes for reducing depressive and anxiety symptoms (i.e. impairment-level outcomes) after ABI and stroke (Stalder-Luthy et al., 2013; Wang et al., 2018). However, a third found no appreciable effect in a TBI-specific population (Gertler et al., 2015). At the level of activity and participation, the evidence is limited, largely due to a lack of studies that include outcome measures at this level (Gertler et al., 2015; Stalder-Luthy et al., 2013), or meta-analyses pooling these results with impairment-level outcomes (Wang et al., 2018).

One possible explanation for the lack of change at the activity and participation level is that these siloed interventions are not combined to target both cognitive *and* emotional difficulties. There is a strong empirically based rationale for doing so; cognition and emotion are fundamentally connected at both a biological and functional level. From a biological perspective, brain regions responsible for cognition and emotion are integrated into coalitions of networks to the extent that many argue they are not separable systems (Pessoa, 2008). Networks that underpin emotional regulation overlap those that underpin the cognitive domains of memory, executive functioning, attention and working memory. For example, the left dorsolateral prefrontal cortex is crucial for depression, but also working memory and executive functions (Chai et al., 2018; Padmanabhan et al., 2019). At a functional level, there is also a clear argument for targeting cognitive and emotional difficulties in tandem. To use a practical example, a person who has difficulty sustaining attention since their injury may feel embarrassed and anxious when they become distracted or lose track during conversations. They may then avoid situations in which they are required to converse in groups or with new people, and so limit their social interactions. Thus, targeting only one component of the individual’s experience (e.g. only the anxiety, or only attention difficulties) may not fully resolve the functional impact issue. Furthermore, our collective experience of delivering cognitive rehabilitation in Australia and UK indicates that ABI survivors do not often distinguish between cognitive and mood problems when they co-occur, making it difficult to faithfully deliver an intervention that only targets one symptom alone.

Interventions that do target both components have been effective in other populations, for example adults with mild cognitive impairment (Aben et al., 2013). The importance and value of holistic, integrated interventions have been championed by many leading researchers and practitioners in the ABI field (e.g. Wilson, 2013), but as research still largely focuses on siloed interventions (i.e. interventions that address only cognitive, or only psychological concerns), there remains a need for high-level evidence to support these claims.

Several trials have evaluated interventions that combine cognitive and psychological components; however, these trials are mostly underpowered by small samples, heterogeneous (e.g. content and length of interventions; sample characteristics) and fail to measure outcomes at multiple levels (impairment, activity, participation, quality of life). A systematic quantitative summary can resolve these limitations and is overdue. As all previous meta-analyses have only investigated the effect of either psychological or cognitive interventions alone (e.g. Cicerone et al., 2019; Rogers et al., 2018; Stalder-Luthy et al., 2013; Wang et al., 2018), our objective was to perform a systematic review and meta-analysis to evaluate the impact of combined psychological and cognitive interventions on outcomes after ABI. Our primary outcomes of interest were those measuring activity, participation and quality of life, and secondary outcomes of interest were those measuring impairment (in cognition, emotion, coping/emotional regulation, fatigue, and self-efficacy).

## Method

This meta-analysis is reported in accordance with the Preferred Reporting Items for Systematic Review and Meta-Analysis (PRISMA) statement (Liberati et al., 2009). The protocol was registered with the International Prospective Register of Systematic Reviews (PROSPERO) on 03 Nov. 2020 (CRD42020207663), where the protocol can be accessed.

### Inclusion and Exclusion Criteria

#### Types of Studies

Eligible studies were randomised controlled trials (RCTs), published in English in peer-reviewed journals.

#### Participants

Participants were required to be adults (aged 16 + years) with a clinical diagnosis of non-progressive ABI (e.g. ischaemic or haemorrhagic stroke, TBI, encephalitis, tumour), at least one-month post-injury. We created this cut-off as our primary outcomes relate to sub-acute and more chronic outcomes. Studies were excluded if participants were primarily those with severe psychiatric conditions such as post-traumatic stress disorder (PTSD) or psychosis, or if the time since injury could not be confirmed. All levels of injury severity were included. Studies focusing on participants with aphasia were included, as long as they met the necessary intervention criteria.

#### Types of Interventions and Controls

Due to the broad range of interventions used to address the cognitive and psychological consequences of ABI and the inconsistent terminology used to report them, definitions for each intervention subtype were established to ensure consistency between reviewers. Psychological interventions were defined as those that use psychotherapeutic strategies (O'Malley et al., 2016) to address issues of emotions, thought or behaviour (Hickey et al., 2019), such as cognitive behavioural therapy (CBT) (Beck, 1976) and acceptance and commitment therapy (ACT) (Hayes et al., 1999). Cognitive interventions were defined as those that aim to restore or compensate for cognitive impairments (Wong et al., 2014), such as training and use of compensatory memory aids (Rees et al., 2007; Sohlberg et al., 2007), meta-cognitive strategy instruction (Kennedy et al., 2008), attention retraining (Loetscher et al., 2019) and computerised training (Poulin et al., 2012). Combined interventions were defined as those that clearly combined both cognitive and psychological intervention elements. Interventions were considered combined if they used both cognitive and psychotherapeutic rehabilitation methods to directly target both the cognitive and emotional consequences of ABI, even if one target was more prominent. While combined interventions varied in the degree to which they integrated cognitive and psychological elements, none were excluded due to ‘lack of integration’. Interventions where mindfulness was the only psychological intervention element were not considered combined interventions due to the ambiguity in the treatment target (i.e. mindfulness can be used as a cognitive strategy to improve attention, not necessarily a psychological technique to reduce anxiety). However, if the psychological element involved mindfulness in the context of a psychotherapeutic framework with a clear psychological target (e.g. mindfulness-based stress reduction therapy), that was considered a combined intervention.

We excluded interventions targeting behaviours of concern (such as socially inappropriate behaviour or aggression), social cognition or interventions limited to focal cognitive deficits (such as visual neglect and language difficulties). The latter exclusion was applied because we expected that combined interventions would be most applicable for cognitive impairments in domains that are underpinned by diffuse brain networks (i.e. attention/working memory, memory and executive function) which overlap with the networks that underpin emotion regulation. While social cognition is underpinned by these networks, our decision to exclude this domain was primarily based on the fact that social cognition interventions are inherently combined rather than solely cognitive, due to the nature of the domain (i.e. they usually contain psychological, behavioural and cognitive elements). Also, the research into social cognition interventions is less developed, and currently, there is insufficient information to clearly hypothesise whether social cognitive and emotion perception elements would interact with psychological therapies in the same way as non-social cognitive interventions. It was decided that including these interventions would introduce additional uncertainty in the data set of an already heterogeneous set of studies. The exclusion of interventions targeting behaviours of concern was based on a similar rationale; we considered these interventions as belonging to their own category of ‘behavioural interventions’ rather than cognitive or psychological interventions. However, a similar meta-analysis examining combined cognitive and behavioural interventions would be warranted. Finally, interventions such as music therapy, yoga, physical exercise and dual-task training interventions in which physical exercise was one component were also excluded, despite their target often being cognition. This decision is in line with most systematic reviews of cognitive rehabilitation interventions, which have applied similar exclusions (e.g. das Nair et al., 2016). All pharmacological interventions were excluded.

No restrictions were made regarding intervention variables such as duration, frequency or delivery method (e.g. group, one-on-one). If a trial evaluated several intervention conditions of which some were not eligible (e.g. pharmacological), only the data from the eligible conditions were included. No criteria were set for the nature of the control condition. However, controls were grouped into passive (waitlist), treatment as usual and active (an alternative intervention) for statistical analysis. If multiple possible intervention or control conditions were available within one study, the most appropriate one for this meta-analysis was selected; this occurred on only two occasions. The key criterion for selecting the appropriate intervention group was that the main elements consisted of cognitive and psychological techniques to address difficulties in these areas (e.g. we chose a group-based intervention that combined meta-cognitive skills training with emotion regulation and coping skills over an individual intervention that focused on more functional difficulties such as learning meta-cognitive strategies to improve cooking skills). Decision-making regarding the selection of an appropriate control condition was more pragmatic. We chose a psychoeducation condition over a cognitive intervention, as we hope to include the cognitive intervention as treatment group in a future meta-analysis.

#### Outcome Measures

Primary outcomes of interest were those relating to the domains of activity, participation, and quality of life, as measured by any validated tool. Secondary outcomes of interest were those at the impairment level, including cognition (i.e. cognitive test performance or subjective cognitive function questionnaires), emotion, coping/emotional regulation, fatigue and self-efficacy, as measured by any validated tool. We used the ICF framework to classify outcomes and added in quality of life. Each study could contribute to one or more outcome domains. For studies reporting more than one measurement tool for an outcome domain (e.g. multiple measures of mood), all outcomes were included. However, the effect sizes from multiple-endpoint studies are unlikely to be independent, because the same participants are involved. Unfortunately, primary studies do not routinely include information on how to estimate the degree of dependence (Tipton, 2015). In our study, the common method of applying a correlation of 1.0 was therefore adopted (Cheung, 2019). While this conservative correlation may underestimate the precision of the summary effect, this is offset by the gains in precision made from taking into account all of the data available in the analyses.

We classified outcomes according to whether they were collected immediately post-treatment (within 1 month of intervention end) and longer-term follow-up (more than 1 month after intervention end). For studies that assessed outcomes at multiple post-intervention timepoints, we chose the follow-up time point most distal to the intervention finish date. This was considered most meaningful in terms of assessing maintenance of intervention effects.

### Search Methods for Identification of Studies and Data Extraction

Search strategies were developed in collaboration with an experienced academic librarian. Five databases were systematically searched from inception to 17 Aug. 2022, MEDLINE, PsycINFO, EMBASE, CINAHL and Cochrane Library, each with an individualised search strategy. Briefly, each contained keywords and Medical Subject Heading (MeSH) terms for four concepts, (i) population (acquired brain injury), (ii) intervention type (cognitive, psychological or combined), (iii) intervention target (cognition or emotion) and (iv) study design (randomised controlled trials). The full search strategy for MEDLINE can be found in Appendix 1. The search was restricted to studies published in English, with no restrictions on year of publication.

The database search results were first de-duplicated in Endnote before being uploaded onto Covidence systematic review software for screening (Borenstein et al., 2013). All stages of the screening process (title/abstract and full text) were independently conducted by two reviewers (AD and either LL, JL, KB or JR), and any discrepancies were resolved by a third reviewer (DW).

One reviewer extracted the data for each included study (AD), and accuracy of extraction was verified by DW. Available data concerning study design, participant and intervention characteristic and outcomes at baseline and post-intervention were collected for included studies (Table 1). When possible, intervention information was extracted in line with the TIDIeR checklist (Hoffmann et al., 2014).

**Table 1:** Key characteristics of each study included in the meta-analysis

Author (year)	*n*	Sample (injury type, mean age (SD), % female, mean time since injury (SD); note all at least 1 month post injury)	Delivery mode (individual/group)	Treatment dose	Outcome measures	Outcome time points	
						Immediate	Follow-up
Assonov (2021)	70	TBI, 46.44 (7.67), 3% female, 6 y [5–6] (median and Q1-Q3)	Individual	6 h, 6 weeks	CD-RISC, MoCA, NSI, HADS, PCL-5, PANAS, CQLS	Y	N
Cantor et al. (2014)	98	TBI, 45.3 y (12.7), 63% female, 12.6 y (14.1)	Both	90 h, 12 weeks	BADS, BDI, DERS, FrsBE, Life-3, POPS, PSI STAI	Y	N
Cicerone et al. (2008)	69	TBI, 36.6 y (11.8), 31.5% female, 49.6 m (76.5)	Both	240 h, 16 weeks	NP, PSE, PQOL, CIQ	Y	24 weeks
Cooper et al. (2017)	66	TBI, 31 y (8.3), 7.6% female, 306.6 d (193.2)	Both	42 h, 6 weeks	KBCI, PASAT, SCL90-R	Y	18 weeks
Exner et al. (2022)	62	ABI, 45.6 y (11.1), 31.5% female, 26.1 m (34.9)	Individual	28 h, 36 weeks (mean weeks)	ADFIQ, CIQ, SCL90-R, SEIQoL	Y	N (combined follow-up data across treatment groups)
Nguyen et al. (2017)	24	TBI, 43.8 y (13.0), 33.3% female, 1390.2 d (1671.2)	Individual	8 h, 8 weeks	BFI, FSS, HADS	Y	8 weeks
Nguyen et al. (2019)	15	Stroke, 48.8 y (13.6), 26.7% female, 21.6 m (16.1)	Individual	8 h, 8 weeks	BFI, FSS, HADS, SF-36	Y	8 weeks
Ownsworth et al. (2008)	12	ABI, 43.9 y (12.6), 44.7% female, 5.3 y (3.9)	Group	24 h, 8 weeks	BICRO-39, COPM	Y	Y (weeks post-intervention missing)
Rytter et al. (2019)	89	TBI, missing^a^, 66% female, 26.9 m (16.3)	Both	254 h, 22 weeks	MDI, MFI, RPQ, SF-36	Y	24 weeks
Tiersky et al. (2005)	29	TBI, 46.6 y (10.5), 55% female, 6.25 y (6.0)	Individual	55 h, 11 weeks	CIQ, CRI, PASAT, RAVLT, SCL90-R	N	Y (mean of immediate, 4 and 12-week)
Tornås et al. (2016)	70	ABI, 42.9 y (13.0), 45.1% female, 8.1 y (9.4)	Group	16 h, 8 weeks	BRIEF-A, BREQ, DEX, HSCL-25, QOLIBRI	Y	24 weeks
Urech et al. (2020)	25	ABI, 48.3 y (10.4), 56% female, 14.1 m (13.4)	Missing	1 year (mean 20 sessions)*	ADS, BDI, MFS, ERSQ, WHOQoL	Y	24 weeks
Ymer et al. (2021)	51	ABI, 48.7 y (14.0), 39% female, 62.1 m (62.3)	Individual, telehealth offered	8 h, 8 weeks	BFI, HADS, FSS, SEMBI, SF-36	Y	16 weeks

Study details of included studies, including injury type, mean age and SD, % female, mean time since injury, intervention delivery mode, total intervention hours and total intervention duration, outcome measures used in this review and timepoints of data collected for this review. Follow-up is measured as time since intervention end and was collected at most distal timepoint

*Both* individual and group therapy formats were used, *Missing* information not provided in paper, *SD* standard deviation, ‘*Y*’ yes, ‘*N*’ no, *ADFIQ* Aachen Daily Functioning Item Bank Questionnaire, *ADS* Acceptance of Disability Scale, *BADS* Behavioral Assessment of the Dysexecutive Syndrome, *BDI* Beck Depression Inventory, *BFI* Brief Fatigue Inventory, *BRIEF-A* Behavior Rating Inventory of Executive Function – Adult Version, *BREQ* Brain Injury Rehabilitation Trust Regulation of Emotions Questionnaire, *CD-RISC* Connor-Davidson Resilience Scale, *CRI* Coping Response Inventory, *CIQ* Community Integration Questionnaire, *CQLS* Chablan Quality of Life Scale, *DERS* Difficulties in Emotion Regulation Scale, *DEX* Dysexecutive Questionnaire, *ERSQ* Emotion-Regulation Skills Questionnaire, *FrsBE* Frontal Systems Behavior Scale, *FSS* Fatigue Severity Scale, *HADS* Hospital Anxiety and Depression Scale, *KBCI* Key Behavior Change Inventory, *MDI* Major Depression Inventory, *MFI* Multidimensional Fatigue Inventory, *MFS* Mental Fatigue Scale, *MoCA* Montreal Cognitive Assessment, *NP* Neuropsychological tests, *NSI* Neurobehavioural Symptom Inventory, *PASAT* Paced Auditory Serial Addition Test, *PCL-5* Post-traumatic Stress Disorder Checklist, *RAVLT* Rey Auditory Verbal Learning Task, *RPQ* Rivermead Post Concussion Questionnaire, *POPS* Participation Objective, Participation Subjective, *PQOL* Perceived Quality of Life Scale, *PSE* Perceived self-efficacy for the management of symptoms was adapted from a measure developed for people with chronic medical disability, *PSI* Problem Solving Inventory, *QOLIBRI* Quality of Life after Brain Injury, *SEIQoL* Schedule for the Evaluation of Individual Quality of Life, *SEMBI* Self Efficacy for Managing Brain Injury Questionnaire, adapted from the Stanford Self-Efficacy for managing Chronic Disease 6-item Scale, *SCL90-R* Symptom Checklist-90-Revised, SF-36 36-Item Short Form Survey, *STAI* State-Trait Anxiety Inventory, *WHOQoL* The World Health Organisation Quality of Life

*total treatment hours not provided

^a^Provided age of participants in age brackets, with *n* participants in each bracket so could not calculate mean and SD

### Quality Assessment

Methodological quality of included studies was assessed using the PEDro-P scale, which is derived from the original Physiotherapy Evidence Database (PEDro) Scale trial rating system (Maher et al., 2003). Results of risk of bias were reported according to standard qualitative descriptors (≤ 3 poor quality, 4–5 fair quality and ≥ 6 high quality) (Maher et al., 2003). AD assessed risk of bias for each included study, and the accuracy was checked by DW.

### Quality of Evidence

The overall quality of evidence of the studies was evaluated using the grading of recommendation, assessment, development and evaluation (GRADE) approach (The GRADE Working Group, 2013). AD evaluated the quality of evidence, and the accuracy was checked by DW. Results were reported as an overall score (e.g. high, moderate, low or very low quality) and then discussed by domain (risk of bias, inconsistency, indirectness, imprecision and publication bias).

### Quantitative Analysis

Extracted data was analysed using comprehensive meta-analysis (CMA). A random effects model was used to compute effect size estimate Hedge’s *g*. Hedge’s *g* is a variation of Cohen’s *d* that corrects for small sample sizes, which were common in the included studies. The magnitude of effect size was categorised according to Cohen’s descriptors of *small* (≥ 0.2), *medium* (≥ 0.5) and *large* (≥ 0.8) (Cohen, 1988). All data collected were continuous. Summary effect sizes were calculated based on baseline and post-intervention mean, standard deviation and sample size for treatment and control groups. For all summary effect sizes, 95% confidence intervals (CI) were provided. Where data were missing (e.g. mean or standard deviations), these were requested from the authors. If baseline data were not obtained, group comparisons of post-intervention values only were conducted.

Heterogeneity was assessed with the *Q* (the distribution of observed effects) and tau (the absolute variance of true effects) statistics (Borenstein et al., 2017). Risk of publication bias was quantitatively assessed using Egger’s regression test (two-tailed *p* value) and qualitatively assessed by examining funnel plot asymmetry.

Subgroup analyses were planned at the level of quality of life, participation, activity and impairment (divided into cognition, mood, coping/emotional regulation, fatigue and self-efficacy). Data were analysed at immediate post-intervention and follow-up timepoints separately. Moderator analyses were conducted to explore the extent to which between-study heterogeneity was explained by the variables of control type, injury type, severity and chronicity, intervention delivery mode (e.g. *individual-only*, *group-only* or *both* formats combined) and treatment dose. Sensitivity analysis was performed that excluded low-quality studies. Subgroup and moderator analyses were only performed when the 5-study minimum criterion was met (Jackson & Turner, 2017).

## Results

### Study Characteristics

**Fig. 1:**
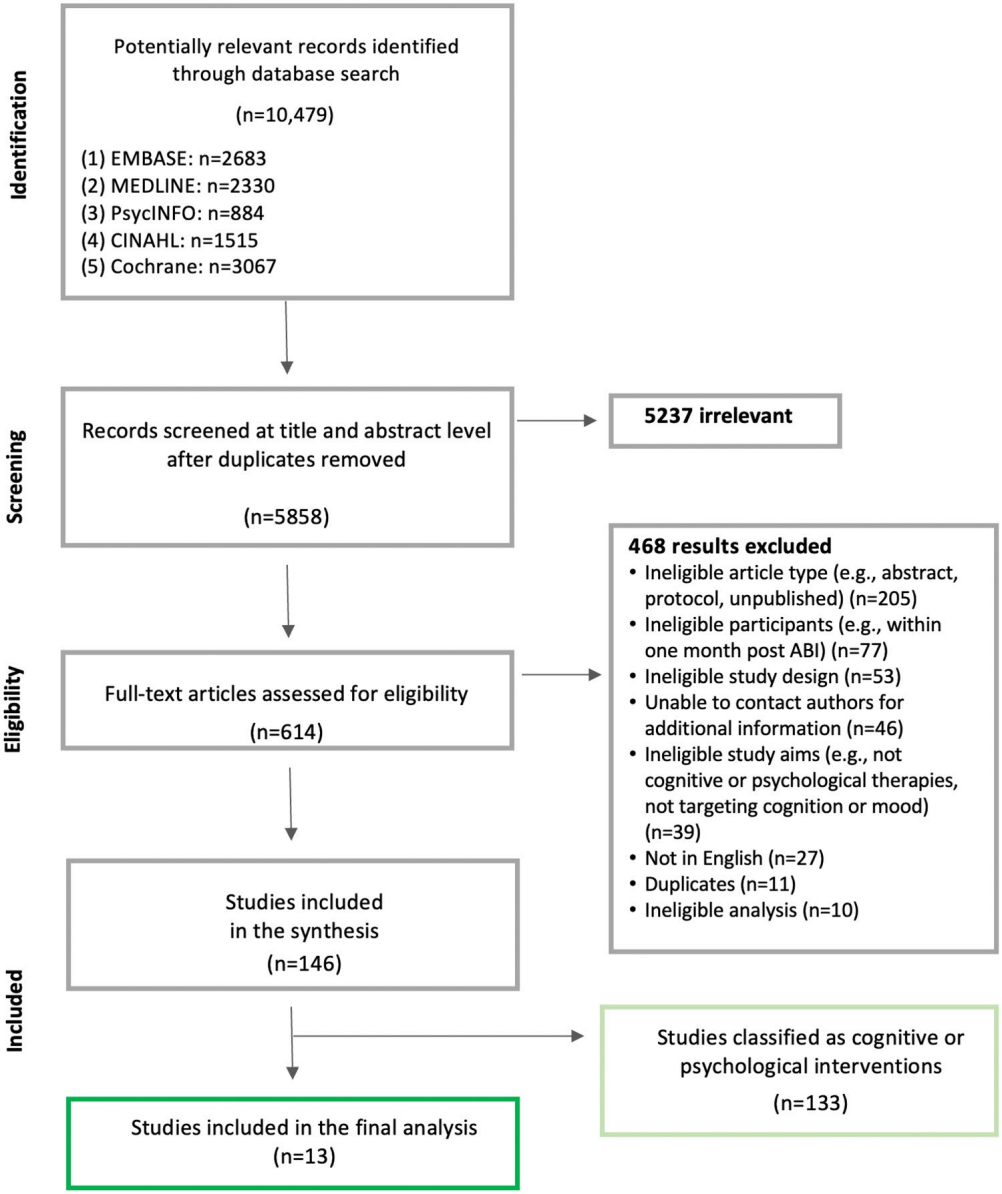
Four-phase PRISMA flow diagram for study selection, detailing identification and screening process for articles included in the review and meta-analysis

Results of the study selection process are depicted in the PRISMA flowchart in Fig. 1. One hundred and forty-eight studies were identified as eligible for inclusion in the planned meta-analyses on cognitive-only and psychological-only interventions. Most studies excluded based on language were Chinese. Forty-six papers were excluded due to no response from authors, with most requests having been regarding clarification of time since injury. Of the 146 eligible studies, 13 were identified as eligible for the current review (Fig. 1). All studies were included in subgroup analyses, though no single study contributed to every analysis as no study measured outcomes in every domain. Four studies were conducted in Australia, four in Europe, four in North America and one in Ukraine.

### Participant Characteristics

Thirteen studies were included in the current meta-analysis, totalling 684 participants (*n* = 341 for intervention group). The sample sizes of the studies ranged from 12 to 98 (mean = 52.3, SD = 27.4; median = 62). Four studies recruited fewer than 30 participants, and eight studies recruited greater than 50 participants. The average age of participants was 43 years (SD = 12.61). Mean time since injury was < 1 year for one study, between 1 and 2 years for four studies and > 3 years for eight studies. Seven studies involved TBI only, one involved stroke only, and five recruited participants with any form of ABI. Of the TBI studies, two included mild severity only, two included mild/moderate severity, and three included any severity.

### Combined and Control Group Interventions

The targets of the combined interventions were broad and generally reflected a desire to improve daily functioning, independence and participation in meaningful activities through targeting mood disturbance and cognitive impairments. Three interventions specifically targeted post-ABI fatigue (Nguyen et al., 2017, 2019; Ymer et al., 2021). The main components of the interventions are detailed in Tables 1 and 2. Consistent across interventions were the inclusion of psychoeducation regarding ABI symptoms, compensatory cognitive strategies (addressing different cognitive deficits, but commonly attention, memory, meta-cognition, organisation, problem solving, fatigue and emotional regulation) and psychotherapeutic strategies to facilitate emotional adjustment after ABI and management of anxiety and depression (most commonly based on a CBT framework) (Table 2). A variety of delivery modes were used: group, individual and combined; in-person and telehealth (Table 1). Nine interventions were administered by either a neuropsychologist or psychologist with experience in ABI, occasionally alongside other clinicians, such as occupational therapists. Of the remaining four, three interventions were delivered by psychologists, but the studies did not detail their experience with ABI populations (Cantor et al., 2014; Cooper et al., 2017; Ownsworth et al., 2008), and one did not report the qualifications of the therapist (Assonov, 2021).

**Table 2:** Psychological and cognitive components of studies’ interventions

Intervention	Psychological component	Cognitive compensatory component	Cognitive restorative component	Other
Assonov (2021)	Psychoeducation and strategy development regarding stress management, emotional control, stress identification and reduction and understanding the connection between emotional state and resilience and ability to regulate emotions	Psychoeducation and strategy development for concerns with concentration, memory, goal setting, cognitive flexibility, problem solving, and understanding connection between emotional state and ability to complete tasks	N/A	
Cantor et al. (2014)	Problem solving training and emotion regulation training (based on CBT)	Support in using external aids such as calendars and to do lists	Attention training (based on Attention Process Training)	
Cicerone et al. (2008)	Emotion regulation, acceptance and awareness of role limitations, and the benefits of the therapeutic milieu (no specific theoretical framework mentioned)	Education regarding metacognition (self-monitoring and self-regulation) and strategies to manage attentional difficulties, planning, organisation, goal setting and social problem solving and how to apply these in daily life	N/A	
Cooper et al. (2017)	Individual therapy targeting anxiety/stress (based on ACT and MBSR) and group therapy targeting psychotherapy for post-concussive symptoms and depressive symptoms (based on CBT)	Compensatory cognitive strategies	Restorative cognitive strategies, and computerised cognitive training based on Attention Process Training-3 (ATP-3)[1]	
Exner et al. (2022)	Emotional adjustment including psychoeducation about the impact of ABI on emotions and life goals, understanding and applying the CBT model using activities and strategies for cognitive reappraisal of self	Strategies to manage attention, memory and executive function difficulties and how to apply these in daily life	N/A	
Nguyen et al. (2017)	Core CBT principles of psychoeducation, behavioural activation, behaviour experiments, modification of unhelpful thinking styles, problem-solving, relaxation and relapse prevention	Cognitive strategies for managing fatigue (e.g. preventing information overload, memory aids, time-pressure management) and sequencing activities to conserve energy	N/A	Physical exercise as part of CBT behavioural activation techniques
Nguyen et al. (2019)	Core CBT principles of psychoeducation, behavioural activation, behaviour experiments, modification of unhelpful thinking styles, problem-solving, relaxation and relapse prevention	Cognitive strategies for managing fatigue (e.g. preventing information overload, memory aids, time-pressure management) and sequencing activities to conserve energy	N/A	Physical exercise as part of CBT behavioural activation techniques
Ownsworth et al. (2008)	Understanding and managing emotional changes, coping and motivation	Development of metacognitive skills (self-awareness and use of compensatory strategies) through group-based psychoeducation, peer and facilitator feedback and goal setting	N/A	
Rytter et al. (2019)	Building insight into the management of stress, anxiety and depression-like symptoms, developing strategies for coping (based on CBT)	Education regarding cognitive difficulties associated with concussion, factors that can potentiate the symptoms or reduce them and strategies to manage mental fatigue	N/A	Physical exercise training aimed at raising the general activity level of the participants
Tiersky et al. (2005)	Increasing the use of effective coping behaviours, reducing levels of stress, teaching skills for preventing relapse (i.e. return of emotional distress) and helping participants to cope with feelings of loss related to decreased cognitive and physical functioning (based on CBT)	Improving attention and concentration skills through strategies such as single task completion, removal of distractions and planning and problem-solving techniques. Improving memory using compensatory strategies, focusing on memory book training	Restorative cognitive techniques were drawn from Attention Process Training	
Tornås et al. (2016)	Emotional regulation module, based on the core concepts from CBT, emphasising the mutual relationship between thoughts, situations and emotions, and how negative self-talk can become ‘automatic’ and interfere with goal achievement. Mindfulness exercises practiced to enhance awareness of the ongoing situation and goals	Goal management training was adapted from Levine and colleagues manual-based protocols [2]. 9 GMT modules were merged into 7, addressing all core concepts of GMT in the same order	N/A	
Urech et al. (2020)	Coping strategies to facilitate emotional adjustment to the consequences of an ABI (adapted from cognitive‚ behavioural, emotion-focused, process-experiential and interpersonal psychotherapies)	Compensatory strategy development (e.g. external memory aids), fatigue management and psychoeducation about ABI symptoms	N/A	
Ymer et al. (2021)	Modifying unhelpful beliefs and behaviours contributing to sleep disturbance and fatigue (based on CBT)	Cognitive strategies for mental fatigue (e.g. breaking down complex tasks, time pressure management, task pacing)	N/A	

*ACT* acceptance and commitment therapy, *CBT* cognitive behavioural therapy, *MBSR* mindfulness-based stress reduction

[1] S. Zickefoose, K. Hux, J. Brown, and K. Wulf, "Let the games begin: a preliminary study using Attention Process Training -3 and Luminosity brain games to remediate attention deficits following traumatic brain injury," *Brain Injury* vol. 27, no. 6, pp. 707–716, 2013

[2] B. Levine, T. A. Schweizer, C. O'Connor, G. Turner, S. Gillingham, and D. T. Stuss, "Rehabilitation of executive functioning in patients with frontal lobe brain damage with goal management training," *Frontiers in human neuroscience,* vol. 5, no. 9, pp. 1‐9, 2011, https://doi.org/10.3389/fnhum.2011.00009

Five studies used active controls (Cicerone et al., 2008; Cooper et al., 2017; Tornås et al., 2016; Urech et al., 2020; Ymer et al., 2021). The content of active controls varied from basic psychoeducation to individual and/or group sessions with clinicians. For the more complex control interventions, they either did not include psychotherapeutic techniques or discuss emotional difficulties (i.e. single siloed intervention), or they did not integrate the different components of the intervention (i.e. a series of siloed interventions). The remaining eight studies were classified as either treatment as usual (TAU) (*k* = 4) or waitlist (*k* = 4). What TAU involved varied depending on the local healthcare system, but permitted engagement with multidisciplinary clinicians (i.e. a series of siloed interventions). It was not reported if waitlist controls were prohibited from engaging with any particular therapies.

The duration of interventions ranged from 6 to 52 weeks (mode = 8 weeks, mean = 13 weeks); this detail was missing from one study (Assonov, 2021). The total hours of intervention ranged from 6 to 254 h (mean = 64.8, SD = 84.8); this detail was missing from one study (Urech et al., 2020). The most frequently omitted TIDieR checklist details related to fidelity of intervention adherence and delivery.

### Outcome Measures

Outcomes were measured at multiple levels within and across studies: quality of life, participation, activity and impairment (secondary outcomes of interest). The categorisation of outcome measures into levels can be found in the Appendix 2. The most commonly assessed level was impairment (*k* = 13), and within this, the domains of emotion (*k* = 12), cognition (*k* = 6), fatigue (*k* = 5), coping/emotional regulation (*k* = 4) and self-efficacy (*k* = 3). Following this was quality of life (*k* = 9), activity (*k* = 7) and participation (*k* = 5). Of the 54 different measurement tools used across the 13 studies, only eight were used in more than one study (BDI, BFI, CIQ, FSS, HADS, SCL-90-R, SF-36 and QOLIBRI (used in ≤ 3 studies)).

### Risk of Bias

**Fig. 2:**
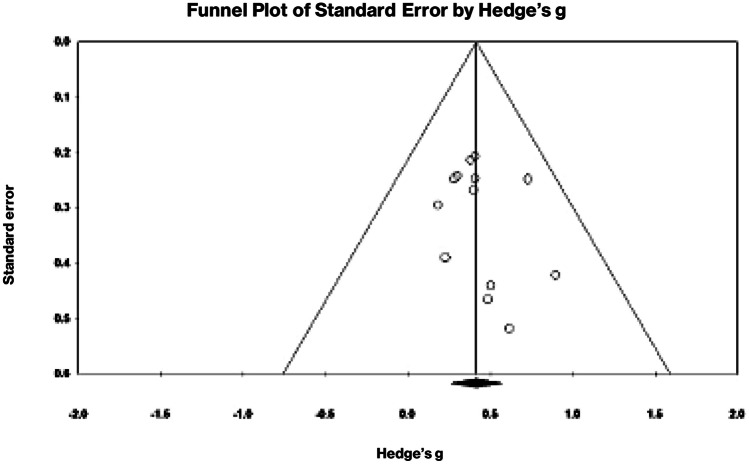
Funnel plot of the overall effect size Hedge’s *g* against standard errors

Overall, the studies were of *high* methodological quality (mean PEDro-P score = 6.8, SD = 0.9, range 5–8) (Table 3). Only one study was classified as *fair quality* (Cooper et al., 2017). The most common risks of bias were not blinding participants or therapists (often not possible due to study design), followed by incomplete reporting of results, and high levels of attrition. Visual inspection of the funnel plot (Fig. 2) and statistical analysis did not reveal significant asymmetry (Egger’s intercept for all outcomes combined was 0.57, *p* = 0.37, two-tailed). This indicates that smaller studies were not more likely to report larger than average effects, i.e. no publication bias was evident.

**Table 3:** Risk of bias ratings (PEDro-P scale)

**Study name**		**Criteria**										
	C1	C2	C3	C4	C5	C6	C7	C8	C9	C10	C11	Total
Assonov (2021)	1	1	1	1	0	0	0	1	1	1	1	7
Cantor et al. (2014)	1	1	1	1	0	1	1	1	1	1	0	8
Cooper et al. (2017)	1	1	0	1	0	0	1	0	1	1	0	5
Cicerone et al. (2008)	1	1	1	1	0	0	1	1	1	1	0	7
Exner et al. (2022)	1	1	1	1	0	0	0	1	1	1	0	6
Nguyen et al. (2017)	1	1	1	1	0	0	1	1	1	1	1	8
Nguyen (2019)	1	1	1	1	0	0	1	1	1	1	1	8
Ownsworth et al. (2008)	1	1	1	1	0	0	1	1	1	0	0	6
Rytter et al. (2019)	1	1	1	1	0	0	1	1	1	1	0	7
Tiersky et al. (2005)	1	1	1	1	0	0	1	0	1	1	0	6
Tornås et al. (2016)	1	1	1	1	1	0	1	1	1	1	0	8
Urech et al. (2020)	1	1	1	1	0	0	0	1	1	1	1	7
Ymer et al. (2021)	1	1	1	0	0	0	1	0	1	1	1	6

PEDro-P rating scale assesses risk of bias using 11 criteria. ‘1’ indicates that a study has met that criterion, ‘0’ indicates criterion not met

*C1* eligibility criteria were specified, not included in overall PEDro-P score, *C2* subjects were randomly allocated to groups, *C3* allocation concealed, *C4* groups were similar at baseline regarding the most important prognostic indicators, *C5* blinding of all subjects, *C6* blinding of all therapists who administered the therapy, *C7* blinding of all assessors who measured at least one key outcome, *C8* measures of at least one key outcome were obtained from more than 85% of the subjects initially allocated to groups, *C9* all subjects for whom outcome measures were available received the treatment or control condition as allocated or, where this was not the case, data for at least one key outcome was analysed by intention to treat, *C10* results of between-group statistical comparisons are reported for at least one key outcome, *C11* study provides both point measures and measures of variability for at least one key outcome

### Overall Efficacy of Combined Interventions

**Fig. 3:**
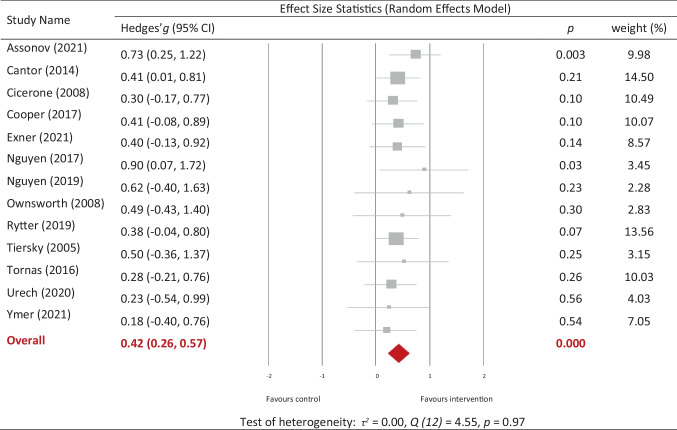
Overall effects of combined interventions on all outcomes combined across timepoints

For all outcomes and timepoints, combined interventions had an overall significant *small-*to*-medium* effect compared to control conditions [*g* = 0.42, (95% CI 0.26, 0.57), *p* < 0.01] (Fig. 3). Heterogeneity was not significant (*p* = 0.97). There was no significant moderating effect of control type (*p* = 0.50), treatment dose (*p* = 0.53), injury type (*p* = 0.22), injury chronicity (*p* = 0.10) or intervention delivery mode (*p* = 0.49). For delivery mode, only *individual-only* and *both* formats were analysed as there was insufficient data to include the *group-only* format. Similarly, for injury type, only ABI and TBI were analysed as there was only one stroke-only study. Sensitivity analysis revealed that quality of study did not have a significant impact on the effect sizes.

### Efficacy of Combined Interventions on Specific Outcomes of Interest Combined Across Timepoints

**Fig. 4:**
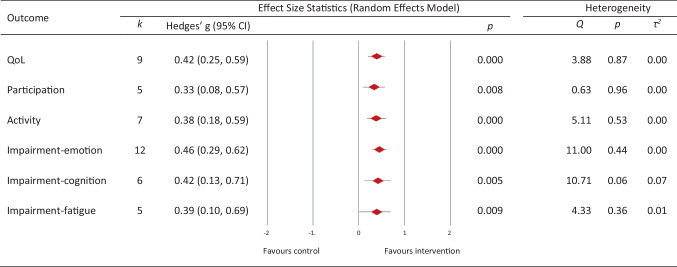
Outcome-specific effects of combined interventions combined across timepoints

*Small*-to-*medium* effects were observed on quality of life (*k* = 9, *g* = 0.42, (95% CI 0.25, 0.59, *p* < 0.0001), participation (*k* = 5, *g* = 0.33, (95% CI, 0.08, 0.57), *p* = 0.0001) and activity (*k* = 7, *g* = 0.38, (95% CI, 0.18, 0.59), *p* < 0.0001) (Fig. 4). At the level of impairment, s*mall*-to-*medium* effects were observed on impairment-cognition (*k* = 6, *g* = 0.42, (95% CI, 0.13, 0.71), *p* = 0.005), impairment-emotion (*k* = 12, *g* = 0.46, (95% CI 0.29, 0.62), *p* < 0.0001) and impairment-fatigue (*k* = 5, *g* = 0.39, (95% CI, 0.1, 0.69, *p* = 0.009). Due to limited data regarding impairment-self-efficacy and impairment-coping/emotional regulation outcomes, these were not meta-analysed.

### Immediate and Follow-up Effect Size Comparisons

**Fig. 5:**
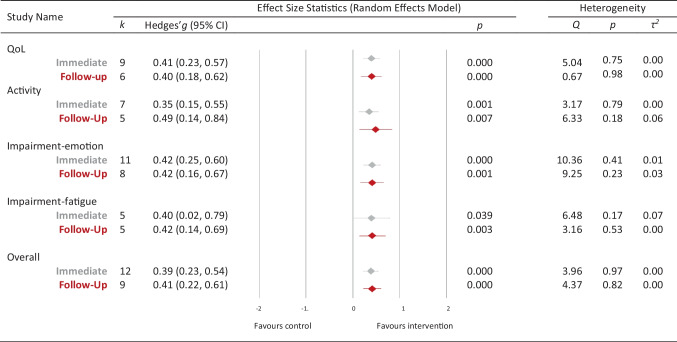
Overall and outcome-specific effects at immediate and follow-up timepoints

All outcomes were significant at both immediate (T1) and follow-up (T2) timepoints (Fig. 5). The overall efficacy of combined interventions immediately post-intervention was *g* = 0.39, which was maintained at follow-up (*g* = 0.41). The same trend was observed for activity (T1, *g* = 0.35; T2, *g* = 0.49), quality of life (T1, *g* = 0.41; T2 *g* = 0.40), impairment-emotion (T1, *g* = 0.42, T2, *g* = 0.42) and impairment-fatigue (T1, *g* = 0.40, T2, *g* = 0.42). Participation and impairment-cognition, self-efficacy and coping/emotional regulation did not have sufficient data to perform these meta-analyses.

### Overall Study Quality

The GRADE framework was used to rate the confidence in the effects of combined interventions on quality of life, participation, activity and impairment outcomes. As all studies were randomised controlled trials, the level of confidence in the effects started at *high*. No points were deducted for imprecision, indirectness, publication bias or risk of bias. Heterogeneity was not significant and moderator analyses did not report a significant effect of study quality on overall or specific outcomes. Based on this, the overall confidence in the effect of combined interventions on all outcomes was rated as *high*.

## Discussion

Despite the growing body of evidence for combined approaches to ABI rehabilitation, no study has systematically evaluated the literature in this field. The current meta-analytic review provides evidence that combined interventions improve outcomes that are typically the most meaningful to ABI survivors. Furthermore, secondary analyses of critical design and implementation features suggest that the efficacy of such interventions appears to be robust across cohorts, settings, delivery methods and time. Importantly, both the sensitivity analysis and GRADE rating provide a confidence in the effects calculated by this meta-analysis. This suggests that more widespread implementation of combined cognitive and psychological interventions may be warranted as part of standard ABI rehabilitation.

### Overall and Outcome-Specific Efficacy

Combined interventions had a *small*-to-*medium* effect on all primary outcomes, which were maintained at follow-up (activity, participation, QoL). Similarly, there was a *small*-to-*medium* effect on secondary outcomes (impairment-emotion, cognition and fatigue), which were again maintained at follow-up. The maintenance effects may speak to the ability of combined interventions to equip individuals with enduring strategies that can be generalised to many areas of their life, enabling them to continue to adapt to changes in environment or needs. That said, the most distal follow-up assessment occurred at 6 months post-intervention, so whether these gains are maintained in the longer-term remains unknown. The results of the current review also demonstrate that combined interventions improve outcomes at all levels, from reducing symptom severity to improving quality of life. While intervention characteristics between studies were variable, effect sizes were similar across domains and studies (i.e. low heterogeneity). This indicates that there may be common elements to the way in which those combined interventions were delivered that led to consistent and successful results, such as their focus on broad, meaningful outcomes. Identifying the ‘active ingredients’ of these interventions should be a key focus of future meta-analyses. Finally, the consistency of effect sizes between included studies (i.e. low heterogeneity) is an important finding. It suggests that despite the small sample size, the current meta-analysis was adequately powered, and high confidence can be placed on the results presented.

### Analysis of Moderating Factors

The low heterogeneity of effect sizes combined with an overall sample of only 13 studies meant that it was difficult to identify statistically significant sub-groups or moderating variables. These results need to be replicated across more studies to confirm this study’s finding of no moderating effects of injury characteristics, intervention characteristics or control group. If replicated, this may have implications for cost–benefit analyses. For example, if no benefit is gained from year-long compared to 6 week-long interventions; this is important to know for healthcare systems that cannot offer lengthy interventions. However, all interventions were a minimum of 6 h and spread across a minimum of 6 weeks, so the efficacy of even briefer interventions is unknown (e.g. single session). It may be that dose response effects would be seen with the inclusion of these much briefer interventions.

### Quality Assessments

The overall quality of studies included in this meta-analysis was *high*, with only one study scoring in the *fair quality* range. However, relying solely on overall quality ratings can mask areas of concern as all studies produced some bias. As such, examining biases by domain is valuable (e.g. selection bias, reporting bias). The most common risk was not blinding participants or therapists to condition. Though this was often not possible due to the studies’ designs, methods for balancing expectation bias were not always applied or reported.

Secondly, three studies (Cooper et al., 2017; Tiersky et al., 2005; Ymer et al., 2021) reported higher than accepted rates of attrition on the Pedro-P scale (Maher et al., 2003). This may speak to the acceptability or feasibility of the interventions, though no obvious common factor connected these studies (e.g. treatment dose, injury type or severity). Thirdly, the PEDro-P scale requires studies to only provide complete reporting of one key outcome. Many studies did not report if they powered their study based on the primary outcome or did not report non-significant findings or measures of variance associated with effect sizes, reflecting possible reporting bias.

### Limitations of Included Studies

Systematically reviewing the current literature highlighted certain limitations. Firstly, no study reported the complete TIDieR checklist for reporting interventions (Hoffmann et al., 2014). This is a common problem in rehabilitation research (Mhizha-Murira et al., 2018; Small et al., 2022). Not only does this make reproducibility difficult, it prevents attempts to analyse the ‘active ingredients’ of interventions through meta-analyses (Small et al., 2022). While these details may have been published in study protocols, none were found despite attempts to do so. The most common details omitted related to the fidelity of intervention adherence and delivery. These aspects of an intervention are crucial to know when evaluating its efficacy; for example, level of homework adherence and therapist competence in reviewing homework both strongly moderate outcomes (e.g. Zelencich et al., 2020). Secondly, results were often reported in a way that made including them in meta-analytic studies difficult. In some circumstances, an analysis based on changes from baseline produces a more efficient and powerful effect than comparison of post-intervention values only, as it removes a component of between-person variability (Deeks et al., 2021). Thus, studies that provide the mean and standard deviations for all timepoints separately (baseline, immediate and follow-ups) allow the meta-analyser to account for pre-post correlations and to calculate summary effect sizes at multiple post-intervention timepoints. Many studies omitted data from one of these time points and/or test statistics for non-significant results. This required authors to be contacted for further information, which, if no response was obtained, resulted in less robust summary effect calculations for some variables. However, we note that calculation of a change score requires measurement of the outcome twice and in practice may be less efficient for outcomes that are unstable or difficult to measure precisely, where the measurement error may be larger than true between-person baseline variability (Deeks et al., 2021).

Other limitations related to the outcome measurement tools used. Firstly, few tools were used in more than one study, despite all interventions sharing similar aims. Moreover, some papers used un-validated tools (such tools were excluded in this review), or tools not validated in a cognitively impaired population. Not validating and adapting measures to suit the needs of those with cognitive impairment is common but risks the tool not measuring its intended construct (Whiting et al., 2015). While there is no consensus regarding which measures best capture change at each outcome level, the heterogeneity of outcome tools makes comparisons difficult, while the use of not appropriately validated tools affects the confidence in conclusions drawn. It would be useful to develop guidelines that detail which outcome tools are most appropriate to use at each level in a cognitively impaired population. Such guidelines exist for psychosocial outcomes in the TBI literature (Honan et al., 2017), but not in stroke.

### Strengths and Limitations of the Current Review and Directions for Future Research

The primary strength of this review is that we were able to categorise and outline effects based on ICF levels (and adding quality of life). This categorisation is rarely performed in meta-analyses, in part because many siloed intervention studies do not measure outcomes at these levels, yet doing so makes the findings relevant and meaningful to people with an ABI, their families and clinicians. However, our ability to investigate the interventions more deeply by performing subgroup analyses was limited due to the small sample size. Understanding the core characteristics of therapists, participants and interventions that lead to symptom reduction and meaningful change in a person’s life is essential to advance the field (Prigatano, 2013) and will be a valuable area to investigate further in future meta-analytic reviews.

The systematic analysis of the current literature highlighted other gaps that could be addressed by future studies. Firstly, this review was not able to directly evaluate the relative efficacy of combined interventions compared to siloed interventions (cognitive or psychological only). Previous meta-analyses of siloed cognitive interventions in non-progressive ABI indicate that while improvements are observed at the level of impairment, there is unclear evidence for improvements at the level of quality of life, participation restriction and activity limitation. Furthermore, evidence to suggest that any gains are maintained over time is variable and uncertain (das Nair et al., 2016; Loetscher et al., 2019; Rogers et al., 2018). As a priority, we are currently undertaking meta-analyses of cognitive-only and psychological-only interventions to further address this question. Future research may also aim to establish the relative efficacy of combined interventions to other areas that were excluded in this review, such as social cognition or behavioural interventions. Secondly, it is not possible to state if there are diagnosis-specific differences in response to combined interventions. This is partly due to the included ABI studies not reporting stroke and TBI cohort data separately, and partly due to the paucity of stroke-only studies (only one of the 13 studies investigated intervention efficacy in a stroke-only population; Nguyen et al., 2019). Given that stroke and TBI studies have shown different benefits from interventions (e.g. Gertler et al., 2015; Wang et al., 2018), it could be useful to investigate this further. Meta-analytic reviews could statistically compare the outcomes of the two populations, ideally taking into consideration any differences in age and diffuse versus focal neuropathology. Thirdly, follow-up periods were generally 4 to 6 months post-intervention, limiting our ability to understand the long-term benefits derived from these interventions. Current studies may indeed have longer-term assessments planned (many included studies are fewer than 5 years old). Fourthly, due to the smaller sample size, it was difficult to identify moderating variables, such as the duration of interventions. Some combined interventions in the included studies were lengthy and would not be feasible to implement in many healthcare settings. Future meta-analyses will hopefully be able to comment more definitively on such relationships. In addition, inter-rater reliability was not calculated as the software system (Covidence) did not keep a record of discrepancies after they had been resolved.

A final point to note is that our review focused on interventions where the cognitive and psychological elements were usually deliberately integrated within a structured or manualised intervention. Another way in which cognitive and psychological interventions could be integrated would be to concurrently run ‘siloed’ cognitive and psychological interventions demonstrated to be effective for improving cognitive function and mood separately, including elements from each into a bespoke intervention tailored to the individual. An interesting direction for future research would be to compare a structured, manualised integrated intervention with individually selected multimodal interventions where components have been selected by the clinician from a suite of existing cognitive or psychological interventions based on the case formulation, as often occurs in clinical practice. It is possible that these clinician-selected interventions may be more effective, however may rely on expert clinicians, with structured interventions more feasible for clinicians with less training or expertise. Additionally, structured interventions can provide a useful starting point that allows the clinician to observe the person’s response to various intervention elements, which they could then follow up in more individually tailored sessions after completing the initial structured intervention.

## Clinical Implications and Conclusions

This meta-analysis provides evidence for the efficacy of combined interventions at all outcome levels that are robust across cohorts and delivery methods. Not only does this suggest that more widespread implementation of combined cognitive and psychological interventions is warranted as part of standard ABI rehabilitation, but it also validates their use in general outpatient settings where populations are often varied in terms of injury type, severity and chronicity, and the delivery may vary between individual and group formats. It appears that effective combined therapy can be completed within a standard eight-session therapy window, suggesting that this approach addresses essential elements of value-based healthcare from both the client and health care system perspective. This review should provide the confidence to proceed with formal evaluation of the cost-effectiveness and socio-economic impact of combined approaches. A potential further cost-effectiveness is that benefits of combined interventions are maintained over a follow-up period of up to 6 months. This consistent maintenance of benefits implies that combined interventions are equipping individuals with durable strategies that allow them to continue to adapt to changes in environment or needs, thus enabling individuals with ABI to be more independent and less reliant on continued healthcare services. Having realised the potential of combined approaches, implementation is encouraged, to enhance clinical outcomes in ABI rehabilitation, continue to develop and refine the therapy approach and inform the identification of active ingredients.

## Data Availability

The data that support the findings of this study and data extraction templates are available from the corresponding author upon request.

## Appendix 1. MEDLINE Search Strategy

1. exp Craniocerebral trauma/ or exp Cerebrovascular disorders/ or exp Brain hypoxia/
2. (traumatic brain injur* or acquired brain injur* or stroke* or post-stroke or poststroke or cerebral vasc* or cerebrovasc*).tw
3. ((brain or cerebr* or head or crani*) adj3 (injur* or hypoxi* or damage* or trauma*)).tw
4. 1 or 2 or 3
5. Psychotherapy/ or Psychotherapy, group/ or Behavior Therapy/ or exp Cognitive behavioral therapy/ or exp cognitive remediation/
6. ((metacognit* or memory or executive function* or executive dysfunction* or attention* or cognit* or psycholog* or problem solv* or psychosocial) adj3 (intervention* or rehabilitat* or therap* or train* or retrain* or re-train* or program* or remediat* or strateg*)).tw.
7. (neuropsycholog* rehabilitat* or psychotherap* or “acceptance and commitment” or motivational interviewing or strategy training or behavio?ral activation or mindfulness or time pressure management or brain train* or stress management training).tw.
8. 5 or 6 or 7
9. Randomized Controlled Trials/
10. random allocation/ or placebos/
11. randomized controlled trial.pt.
12. controlled clinical trial.pt.
13. clinical trial.pt.
14. randomi?ed.tw.
15. Placebo*.tw.
16. randomly.tw.
17. Trial*.tw.
18. groups.tw.
19. 9 or 10 or 11 or 12 or 13 or 14 or 15 or 16 or 17 or 18
20. exp Animals/ not humans.sh.
21. 19 not 20

22. 4 and 8 and

## Appendix 2. Categorisation of outcome measures by level

**Table:**

	QoL	Participation restriction	Activity limitation	Impairment-emotion	Impairment-cognition	Impairment-fatigue	Impairment-self efficacy	Impairment-coping/emotion regulation
ADFIQ			X					
ADS	X							
BADS					X			
BDI				X				
BDI				X				
BFI			X					
BICRO-39 (socialising and productive employment subscales)		X						
BICRO-39 (psychological well-being subscale)				X				
BREQ								X
BRIEF-A			X					
CD-RISC							X	
CES-D				X				
CIQ		X						
COPM		X						
CRI								X
CQLS	X							
DERS								X
DEX			X					
ERSQ								X
FrsBE			X					
FSS						X		
HADS				X				
HSCL-25				X				
KBCI			X					
Life-3	X							
MDI				X				
MFI						X		
MFS						X		
Neuropsychological tests (e.g. PASAT, RAVLT)					X			
NSI					X			
PCL-5				X				
PANAS				X				
PQoL	X							
POPS		X						
PSE							X	
PSI								X
QOLIBRI	X							
RPQ					X			
SCL-90-R				X				
SEMBI							X	
SEQoL	X							
SF-36 item	X							
STAI				X				
WHOQoL	X							

*ADFIQ* Aachen Daily Functioning Item Bank Questionnaire, *ADS* Acceptance of Disability Scale, *BADS* Behavioral Assessment of the Dysexecutive Syndrome, *BDI* Beck Depression Inventory, *BFI* Brief Fatigue Inventory, *BRIEF-A* Behavior Rating Inventory of Executive Function – Adult Version, *BREQ* Brain Injury Rehabilitation Trust Regulation of Emotions Questionnaire, *CD-RISC* Connor-Davidson Resilience Scale, *CRI* Coping Response Inventory, *CIQ* Community Integration Questionnaire, *CQLS* Chablan Quality of Life Scale, *DERS* Difficulties in Emotion Regulation Scale, *DEX* Dysexecutive Questionnaire, *ERSQ* Emotion-Regulation Skills Questionnaire, *FrsBE* Frontal Systems Behavior Scale, *FSS* Fatigue Severity Scale, *HADS* Hospital Anxiety and Depression Scale, *KBCI* Key Behavior Change Inventory, *MDI* Major Depression Inventory, *MFI* Multidimensional Fatigue Inventory, *MFS* Mental Fatigue Scale, *MoCA* Montreal Cognitive Assessment, *NP* Neuropsychological tests, *NSI* Neurobehavioural Symptom Inventory, *PASAT* Paced Auditory Serial Addition Test, *PCL-5* Post-traumatic Stress Disorder Checklist, *RAVLT* Rey Auditory Verbal Learning Task, *RPQ* Rivermead Post Concussion Questionnaire, *POPS* Participation Objective, Participation Subjective, *PQOL* Perceived Quality of Life Scale, *PSE* Perceived self-efficacy for the management of symptoms was adapted from a measure developed for people with chronic medical disability, *PSI* Problem Solving Inventory, *QOLIBRI* Quality of Life after Brain Injury, *SEIQoL* Schedule for the Evaluation of Individual Quality of Life, *SEMBI* Self Efficacy for Managing Brain Injury Questionnaire, adapted from the Stanford Self-Efficacy for managing Chronic Disease 6-item Scale, *SCL90-R* Symptom Checklist-90-Revised, *SF-36* 36-Item Short Form Survey, *STAI* State-Trait Anxiety Inventory, *WHOQoL* The World Health Organisation Quality of Life

## Abbreviations

ACT: Acceptance and commitment therapy
ABI: Acquired brain injury
CBT: Cognitive behavioural therapy
ICF: International Classification of Functioning
PTSD: Post-traumatic stress disorder
QoL: Quality of life
TAU: Treatment as usual
TBI: Traumatic brain injury

## References

[CR1] Aben, L., Heijenbrok-Kal, M. H., van Loon, E. M., Groet, E., Ponds, R. W., Busschbach, J. J., & Ribbers, G. M. (2013). Training memory self-efficacy in the chronic stage after stroke: A randomized controlled trial. *Neurorehabilitation and Neural Repair,**27*(2), 110–117. 10.1177/1545968312455222 22895620 10.1177/1545968312455222

[CR2] Access Economics. (2009). The economic cost of spinal cord injury and traumatic brain injury in Australia. *Report by Access Economics for the Victorian Neurotrauma Initiative. Canberra: Access Economics, 31*.

[CR3] Andrew, N. E., Kilkenny, M., Naylor, R., Purvis, T., Lalor, E., Moloczij, N., & Cadilhac, D. A. (2014). Understanding long-term unmet needs in Australian survivors of stroke. *International Journal of Stroke,**9*(SA100), 106–112. 10.1111/ijs.12325 25042019 10.1111/ijs.12325

[CR4] Anson, K., & Ponsford, J. (2006). Coping and emotional adjustment following traumatic brain injury. *The Journal of Head Trauma Rehabilitation,**21*(3), 248–259. 10.1097/00001199-200605000-00005 16717502 10.1097/00001199-200605000-00005

[CR5] Assonov, D. (2021). Two-step resilience-oriented intervention for veterans with traumatic brain injury: A pilot randomised controlled trial. *Clinical Neuropsychiatry,**18*(5), 247–259. 34984068 10.36131/cnfioritieditore20210503 PMC8696289

[CR6] Beck, A. T. (1976). *Cognitive therapy and the emotional disorders*. International Universities Press.

[CR7] Borenstein, M., Hedges, L., Higgins, J., & Rothstein, H. (2013). Comprehensive meta-analysis version 3 (Version 3). Englewood, NJ: Biostat.

[CR8] Borenstein, M., Hedges, L., Higgins, J., & Rothstein, H. (2017). Basics of meta-analysis: I2 is not an absolute measure of heterogeneity. *Research Synthesis Methods,**8*(1), 5–18. 10.1002/jrsm.1230 28058794 10.1002/jrsm.1230

[CR9] Campbell Burton, C. A., Murray, J., Holmes, J., Astin, F., Greenwood, D., & Knapp, P. (2013). Frequency of anxiety after stroke: A systematic review and meta-analysis of observational studies. *International Journal of Stroke,**8*(7), 545–559. 10.1111/j.1747-4949.2012.00906.x 23013268 10.1111/j.1747-4949.2012.00906.x

[CR10] Cantor, J., Ashman, T., Dams-O’Connor, K., Dijkers, M. P., Gordon, W., Spielman, L., & Oswald, J. (2014). Evaluation of the short-term executive plus intervention for executive dysfunction after traumatic brain injury: a randomized controlled trial with minimization. *Archives of Physical Medicine and Rehabilitation,**95*(1), 1-9.e3. 10.1016/j.apmr.2013.08.005 23988395 10.1016/j.apmr.2013.08.005

[CR11] Chai, W. J., Abd Hamid, A. I., & Abdullah, J. M. (2018). Working memory from the psychological and neurosciences perspectives: a review. *Frontiers in Psychology,**9*, 401. 10.3389/fpsyg.2018.00401 29636715 10.3389/fpsyg.2018.00401 PMC5881171

[CR12] Cheung, M. W.-L. (2019). A guide to conducting a meta-analysis with non-independent effect sizes. *Neuropsychology Review,**29*(4), 387–396. 10.1007/s11065-019-09415-6 31446547 10.1007/s11065-019-09415-6 PMC6892772

[CR13] Cicerone, K. D., Goldin, Y., Ganci, K., Rosenbaum, A., Wethe, J. V., Langenbahn, D. M., & Harley, J. P. (2019). Evidence-based cognitive rehabilitation: systematic review of the literature from 2009 Through 2014. *Archives of Physical Medicine & Rehabilitation,**100*(8), 1515–1533. 10.1016/j.apmr.2019.02.011 30926291 10.1016/j.apmr.2019.02.011

[CR14] Cicerone, K. D., Mott, T., Azulay, J., Sharlow-Galella, M. A., Ellmo, W. J., Paradise, S., & Friel, J. C. (2008). A randomized controlled trial of holistic neuropsychologic rehabilitation after traumatic brain injury. *Archives of Physical Medicine and Rehabilitation,**89*(12), 2239–2249. 10.1016/j.apmr.2008.06.017 19061735 10.1016/j.apmr.2008.06.017

[CR15] Cohen, J. (1988). *Statistical power analysis for the behavioral sciences* (2nd ed.). Lawrence Erlbaum Associates, Publishers.

[CR16] Cooper, D. B., Bowles, A. O., Kennedy, J. E., Curtiss, G., French, L. M., Tate, D. F., & Vanderploeg, R. D. (2017). Cognitive rehabilitation for military service members with mild traumatic brain injury: A randomized clinical trial. *Journal of Head Trauma Rehabilitation,**32*(3), E1–E15. 10.1097/HTR.0000000000000254 27603763 10.1097/HTR.0000000000000254

[CR17] das Nair, R., Cogger, H., Worthington, E., & Lincoln, N. B. (2016). Cognitive rehabilitation for memory deficits after stroke. *Cochrane Database Systematic Reviews,**9*, CD002293. 10.1002/14651858.CD002293.pub3 10.1002/14651858.CD002293.pub3 PMC6457594 27581994

[CR18] Deeks, J., Higgins, J., & Altman, D. (2021). Analysing data and undertaking meta-analyses. In *Cochrane Handbook for Systematic Reviews of Interventions version 6.2 (updated February 2021)*. Online: Cochrane.

[CR19] Deloitte Access Economics. (2013). *The economic impact of stroke in Australia*. Melbourne: National Stroke Foundation.

[CR20] Exner, C., Doering, B. K., Conrad, N., Künemund, A., Zwick, S., Kühl, K., Nestler, S., & Rief, W. (2022). Integrated neuropsychological and cognitive behavioural therapy after acquired brain injury: A pragmatic randomized clinical trial. *Neuropsychological Rehabilitation,**32*(7), 1495–1529. 10.1080/09602011.2021.1908902 33818305 10.1080/09602011.2021.1908902

[CR21] Gertler, P., Tate, R. L., & Cameron, I. D. (2015). Non-pharmacological interventions for depression in adults and children with traumatic brain injury. *Cochrane Database of Systematic Reviews*. 10.1002/14651858.CD009871.pub2 26663136 10.1002/14651858.CD009871.pub2 PMC8761477

[CR22] Hackett, M. L., & Pickles, K. (2014). Part I: Frequency of depression after stroke: An updated systematic review and meta-analysis of observational studies. *International Journal of Stroke,**9*(8), 1017–1025. 10.1111/ijs.12357 25117911 10.1111/ijs.12357

[CR23] Hayes, S. C., Strosahl, K., & Wilson, K. G. (1999). *Acceptance and commitment therapy: An experiential approach to behavior change*. Guilford Press.

[CR24] Hickey, A., Merriman, N. A., Bruen, C., Mellon, L., Bennett, K., Williams, D., & Doyle, F. (2019). Psychological interventions for managing cognitive impairment after stroke. *Cochrane Database of Systematic Reviews*. 10.1002/14651858.Cd01340631483854

[CR25] Hoffmann, T. C., Glasziou, P. P., Boutron, I., Milne, R., Perera, R., Moher, D., & Michie, S. (2014). Better reporting of interventions: template for intervention description and replication (TIDieR) checklist and guide. *BMJ : British Medical Journal,**348*, g1687. 10.1136/bmj.g1687 24609605 10.1136/bmj.g1687

[CR26] Honan, C., McDonald, S., Tate, R., Ownsworth, T., Togher, L., Fleming, J., & Ponsford, J. (2017). Outcome instruments in moderate-to-severe adult traumatic brain injury: recommendations for use in psychosocial research. *Neuropsychological Rehabilitation*. 10.1080/09602011.2017.1339616 28671050 10.1080/09602011.2017.1339616

[CR27] Jackson, D., & Turner, R. (2017). Power analysis for random-effects meta-analysis. *Research Synthesis Methods,**8*, 290–302. 10.1002/jrsm.1240 28378395 10.1002/jrsm.1240 PMC5590730

[CR28] Kennedy, M. R. T., Coelho, C., Turkstra, L., Ylvisaker, M., Moore Sohlberg, M., Yorkston, K., & Kan, P.-F. (2008). Intervention for executive functions after traumatic brain injury: a systematic review, meta-analysis and clinical recommendations. *Neuropsychological Rehabilitation,**18*(3), 257–299. 10.1080/09602010701748644 18569745 10.1080/09602010701748644

[CR29] Liberati, A., Altman, D. G., Tetzlaff, J., Mulrow, C., Gøtzsche, P. C., Ioannidis, J. P., Clarke, M., Devereaux, P. J., Kleijnen, J., & Moher, D. (2009). The PRISMA statement for reporting systematic reviews and meta-analyses of studies that evaluate healthcare interventions: Explanation and elaboration. *BMJ (Clinical research ed.), 339*, b2700. 10.1136/bmj.b2700 10.1136/bmj.b2700 PMC2714672 19622552

[CR30] Lincoln, N. B., Das Nair, R., Bradshaw, L., Constantinescu, C. S., Drummond, A. E. R., Erven, A., … & Morgan, M. (2015). Cognitive Rehabilitation for Attention and Memory in people with Multiple Sclerosis: study protocol for a randomised controlled trial (CRAMMS). *Trials [Electronic Resource], 16*(1). 10.1186/s13063-015-1016-3 10.1186/s13063-015-1016-3 PMC4672565 26643818

[CR31] Loetscher, T., Potter, K. J., Wong, D., & das Nair, R. (2019). Cognitive rehabilitation for attention deficits following stroke. *Cochrane Database of Systematic Reviews*. 10.1002/14651858.CD002842.pub3 31706263 10.1002/14651858.CD002842.pub3 PMC6953353

[CR32] Maher, C. G., Sherrington, C., Herbert, R. D., Moseley, A. M., & Elkins, M. (2003). Reliability of the PEDro Scale for rating quality of randomized controlled trials. *Physical Therapy,**83*(8), 713–721. 10.1093/ptj/83.8.713 12882612

[CR33] Mellon, L., Brewer, L., Hall, P., Horgan, F., Williams, D., & Hickey, A. (2015). Cognitive impairment six months after ischaemic stroke: A profile from the ASPIRE-S study. *BMC Neurology,**15*, 31. 10.1186/s12883-015-0288-2 25879880 10.1186/s12883-015-0288-2 PMC4359388

[CR34] Mhizha-Murira, J., Drummond, A., Klein, O., & dasNair, R. (2018). Reporting interventions in trials evaluating cognitive rehabilitation in people with multiple sclerosis: A systematic review. *Clinical Rehabilitation,**32*, 243–254. 10.1177/0269215517722583 28828902 10.1177/0269215517722583

[CR35] Nguyen, S., McKay, A., Wong, D., Rajaratnam, S. M., Spitz, G., Williams, G., & Ponsford, J. L. (2017). Cognitive behavior therapy to treat sleep disturbance and fatigue after traumatic brain injury: a pilot randomized controlled trial. *Archives of Physical Medicine and Rehabilitation,**98*(8), 1508-1517.e1502. 10.1016/j.apmr.2017.02.031 28400181 10.1016/j.apmr.2017.02.031

[CR36] Nguyen, S., Wong, D., McKay, A., Rajaratnam, S. M. W., Spitz, G., Williams, G., & Ponsford, J. L. (2019). Cognitive behavioural therapy for post-stroke fatigue and sleep disturbance: a pilot randomised controlled trial with blind assessment. *Neuropsychological Rehabilitation,**29*(5), 723–738. 10.1080/09602011.2017.1326945 28521579 10.1080/09602011.2017.1326945

[CR37] O’Malley, L., Bonetti, D. L., Adair, P., Jervøe-Storm, P.-M., & Preshaw, P. M. (2016). Psychological interventions for improving adherence to oral hygiene instructions in adults with periodontal diseases. *Cochrane Database of Systematic Reviews*. 10.1002/14651858.cd012049 26928225 10.1002/14651858.CD005097.pub3 PMC10641657

[CR38] Ownsworth, T., Fleming, J., Shum, D., Kuipers, P., & Strong, J. (2008). Comparison of individual, group and combined intervention formats in a randomized controlled trial for facilitating goal attainment and improving psychosocial function following acquired brain injury. *Journal of Rehabilitation Medicine,**40*(2), 81–88. 10.2340/16501977-0124 18509570 10.2340/16501977-0124

[CR39] Padmanabhan, J. L., Cooke, D., Joutsa, J., Siddiqi, S. H., Ferguson, M., Darby, R. R., & Fox, M. D. (2019). A human depression circuit derived from focal brain lesions. *Biological Psychiatry,**86*(10), 749–758. 10.1016/j.biopsych.2019.07.023 31561861 10.1016/j.biopsych.2019.07.023 PMC7531583

[CR40] Pessoa, L. (2008). On the relationship between emotion and cognition. *Nature Reviews Neuroscience,**9*(2), 148–158. 10.1038/nrn2317 18209732 10.1038/nrn2317

[CR41] Poulin, V., Korner-Bitensky, N., Dawson, D. R., & Bherer, L. (2012). Efficacy of executive function interventions after stroke: a systematic review. *Opics in Stroke Rehabilitation,**19*(2), 158–171. 10.1310/tsr1902-158 22436364

[CR42] Prigatano, G. P. (2013). Challenges and opportunities facing holistic approaches to neuropsychological rehabilitation. *NeuroRehabilitation,**32*(4), 751–759. 10.3233/nre-130899 23867401 10.3233/NRE-130899

[CR43] Rabinowitz, A. R., & Levin, H. S. (2014). Cognitive sequelae of traumatic brain injury. *Psychiatric Clinics of North America,**37*(1), 1–11. 10.1016/j.psc.2013.11.004 24529420 10.1016/j.psc.2013.11.004 PMC3927143

[CR44] Rees, L., Marshall, S., Hartridge, C., Mackie, D., & Weiser, M. (2007). Cognitive interventions post acquired brain injury. *Brain Injury,**21*(2), 161–200. 10.1080/02699050701201813 17364530 10.1080/02699050701201813

[CR45] Rogers, J. M., Foord, R., Stolwyk, R. J., Wong, D., & Wilson, P. H. (2018). General and domain-specific effectiveness of cognitive remediation after stroke: Systematic literature review and meta-analysis. *Neuropsychology Review,**28*(3), 285–309. 10.1007/s11065-018-9378-4 30006801 10.1007/s11065-018-9378-4

[CR46] Rytter, H. M., Westenbaek, K., Henriksen, H., Christiansen, P., & Humle, F. (2019). Specialized interdisciplinary rehabilitation reduces persistent post-concussive symptoms: A randomized clinical trial. *Brain Injury,**33*(3), 266–281. 10.1080/02699052.2018.1552022 30500267 10.1080/02699052.2018.1552022

[CR47] Small, R., Wilson, P., Wong, D., & Rogers, J. M. (2022). Who, what, when, where, why and how: a systematic review of the quality of post-stroke cognitive rehabilitation protocols. *Annals of Physical and Rehabilitation Medicine*. 10.1016/j.rehab.2021.101623 34933125 10.1016/j.rehab.2021.101623

[CR48] Sohlberg, M. M., Kennedy, M., Avery, J., Coelho, C., Turkstra, L., Ylvisaker, M., & Yorkston, K. (2007). Evidence-based practice for the use of external aids as a memory compensation technique. *Journal of Medical Speech - Language Pathology,**15*, xv+.

[CR49] Stalder-Luthy, F., Messerli-Burgy, N., Hofer, H., Frischknecht, E., Znoj, H., & Barth, J. (2013). Effect of psychological interventions on depressive symptoms in long-term rehabilitation after an acquired brain injury: A systematic review and meta-analysis. *Archives of Physical Medicine & Rehabilitation,**94*(7), 1386–1397. 10.1016/j.apmr.2013.02.013 23439410 10.1016/j.apmr.2013.02.013

[CR50] Stolwyk, R., Mihaljcic, T., Wong, D., Chapman, J., & Rogers, J. (2021). Post-stroke cognitive impairment impacts activity and participation outcomes: A systematic review and meta-analysis. *Stroke,**52*, 748–760. 10.1161/STROKEAHA.120.032215 33493048 10.1161/STROKEAHA.120.032215

[CR51] Taylor, L., Mhizha-Murira, J., Smith, L., Potter, K., Wong, D., Evangelou, N., & Dais Nair, R. (2021). Memory rehabilitation for people with multiple sclerosis. *Cochrane Database of Systematic Reviews*. 10.1002/14651858.cd008754.pub4 34661282 10.1002/14651858.CD008754.pub4 PMC8521643

[CR52] The GRADE Working Group. (2013). *GRADE handbook for grading quality of evidence and strength of recommendations* (H. Schünemann, J. Brożek, G. Guyatt , & A. Oxman Eds.).

[CR53] The World Health Organisation. (2002). *Towards a common language for functioning, disability and health: ICF The International Classification of Functioning, Disability and Health*. Retrieved February 1, 2021, from https://cdn.who.int/media/docs/default-source/classification/icf/icfbeginnersguide.pdf

[CR54] Tiersky, L. A., Anselmi, V., Johnston, M. V., Kurtyka, J., Roosen, E., Schwartz, T., & Deluca, J. (2005). A trial of neuropsychologic rehabilitation in mild-spectrum traumatic brain injury. *Archives of Physical Medicine and Rehabilitation,**86*(8), 1565–1574. 10.1016/j.apmr.2005.03.013 16084809 10.1016/j.apmr.2005.03.013

[CR55] Tipton, E. (2015). Small sample adjustments for robust variance estimation with meta-regression. *Psychological Methods,**20*(3), 375–393. 10.1037/met0000011 24773356 10.1037/met0000011

[CR56] Tornås, S., Løvstad, M., Solbakk, A. K., Schanke, A. K., & Stubberud, J. (2016). Goal management training combined with external cuing as a means to improve emotional regulation, psychological functioning, and quality of life in patients with acquired brain injury: A randomized controlled trial. *Archives of Physical Medicine and Rehabilitation,**97*(11), 1841-1852.e1843. 10.1016/j.apmr.2016.06.014 27424292 10.1016/j.apmr.2016.06.014

[CR57] Urech, A., Krieger, T., Frischknecht, E., Stalder-Luthy, F., Grosse Holtforth, M., Muri, R. M., & Hofer, H. (2020). An integrative neuro-psychotherapy treatment to foster the adjustment in acquired brain injury patients-a randomized controlled study. *Journal of Clinical Medicine,**9*(6), 02. 10.3390/jcm9061684 10.3390/jcm9061684 PMC7355481 32498240

[CR58] Wang, S. B., Wang, Y. Y., Zhang, Q. E., Wu, S. L., Ng, C. H., Ungvari, G. S., & Xiang, Y. T. (2018). Cognitive behavioral therapy for post-stroke depression: a meta-analysis. *Journal of Affective Disorders,**235*, 589–596. 10.1016/j.jad.2018.04.011 29704854 10.1016/j.jad.2018.04.011

[CR59] Whiting, D. L., Deane, F. P., Ciarrochi, J., McLeod, H. J., & Simpson, G. K. (2015). Validating measures of psychological flexibility in a population with acquired brain injury. *Psychological Assessment,**27*(2), 415–423. 10.1037/pas0000050 25419644 10.1037/pas0000050

[CR60] Wilson, B. A. (2013). Neuropsychological rehabilitation: State of the science. *South African Journal of Psychology,**43*(3), 267–277. 10.1177/0081246313494156

[CR61] Wong, D., McKay, A., & Stolwyk, R. (2014). Delivery of psychological interventions by clinical neuropsychologists: Current practice in Australia and implications for training. *Australian Psychologist,**49*(4), 209–222. 10.1111/ap.12061

[CR62] Ymer, L., McKay, A., Wong, D., Frencham, K., Grima, N., Tran, J., … & Ponsford, J. (2021). Cognitive behavioural therapy versus health education for sleep disturbance and fatigue after acquired brain injury: a pilot randomised trial. *Annals of Physical and Rehabilitation Medicine, 64*(5). 10.1016/j.rehab.2021.101560 10.1016/j.rehab.2021.101560 34311119

[CR63] Zelencich, L., Wong, D., Kazantzis, N., McKenzie, D., Downing, M., & Ponsford, J. (2020). Predictors of anxiety and depression symptom improvement in CBT adapted for traumatic brain injury: pre/post injury and therapy process factors. *Journal of the International Neuropsychological Society,**26*(Special issue 1), 97–107. 10.1017/S1355617719000791 31983372 10.1017/S1355617719000791

